# Adapting the Wii Fit Balance Board to Enable Active Video Game Play by Wheelchair Users: User-Centered Design and Usability Evaluation

**DOI:** 10.2196/rehab.8003

**Published:** 2018-03-06

**Authors:** Mohanraj Thirumalai, William B Kirkland, Samuel R Misko, Sangeetha Padalabalanarayanan, Laurie A Malone

**Affiliations:** ^1^ Department of Health Services Administration University of Alabama at Birmingham Birmingham, AL United States; ^2^ UAB/Lakeshore Research Collaborative School of Health Professions University of Alabama at Birmingham Birmingham, AL United States; ^3^ Engineering and Innovative Technology Department University of Alabama at Birmingham Birmingham, AL United States

**Keywords:** sedentary lifestyle, video games, active video gaming, Wii Fit, equipment design, physical activity, exercise, wheelchairs, physical disability, rehabilitation, usability

## Abstract

**Background:**

Active video game (AVG) playing, also known as “exergaming,” is increasingly employed to promote physical activity across all age groups. The Wii Fit Balance Board is a popular gaming controller for AVGs and is used in a variety of settings. However, the commercial off-the-shelf (OTS) design poses several limitations. It is inaccessible to wheelchair users, does not support the use of stabilization assistive devices, and requires the ability to shift the center of balance (COB) in all directions to fully engage in game play.

**Objective:**

The aim of this study was to design an adapted version of the Wii Fit Balance Board to overcome the identified limitations and to evaluate the usability of the newly designed adapted Wii Fit Balance Board in persons with mobility impairments.

**Methods:**

In a previous study, 16 participants tried the OTS version of the Wii Fit Balance Board. On the basis of observed limitations, a team of engineers developed and adapted the design of the Wii Fit Balance Board, which was then subjected to multiple iterations of user feedback and design tweaks. On design completion, we recruited a new pool of participants with mobility impairments for a larger study. During their first visit, we assessed lower-extremity function using selected mobility tasks from the International Classification of Functioning, Disability and Health. During a subsequent session, participants played 2 sets of games on both the OTS and adapted versions of the Wii Fit Balance Board. Order of controller version played first was randomized. After participants played each version, we administered the System Usability Scale (SUS) to examine the participants’ perceived usability.

**Results:**

The adapted version of the Wii Fit Balance Board resulting from the user-centered design approach met the needs of a variety of users. The adapted controller (1) allowed manual wheelchair users to engage in game play, which was previously not possible; (2) included Americans with Disabilities Act-compliant handrails as part of the controller, enabling stable and safe game play; and (3) included a sensitivity control feature, allowing users to fine-tune the controller to match the users’ range of COB motion. More than half the sample could not use the OTS version of the Wii Fit Balance Board, while all participants were able to use the adapted version. All participants rated the adapted Wii Fit Balance Board at a minimum as “good,” while those who could not use the OTS Wii Fit Balance Board rated the adapted Wii Fit Balance Board as “excellent.” We found a significant negative correlation between lower-extremity function and differences between OTS and adapted SUS scores, indicating that as lower-extremity function decreased, participants perceived the adapted Wii Fit Balance Board as more usable.

**Conclusions:**

This study demonstrated a successful adaptation of a widely used AVG controller. The adapted controller’s potential to increase physical activity levels among people with mobility impairments will be evaluated in a subsequent trial.

**Trial Registration:**

ClinicalTrials.gov NCT02994199; https://clinicaltrials.gov/ct2/show/NCT02994199 (Archived by WebCite at http://www.webcitation.org/6xWTyiJWf)

## Introduction

Active video games (AVGs), or exergames, are video games that require players to perform substantial body movement for game play, unlike conventional video games, which use remote control push buttons or joysticks. Video game controllers are peripheral devices through which users interact with gaming environments. There are multiple commercially available gaming consoles, and they include a variety of game controllers. Nintendo Wii is a popular gaming console and can connect to the Nintendo Wii Fit Balance Board game controller (Nintendo of America Inc, Redmond, WA, USA). The Wii Fit Balance Board can be used with a variety of AVGs that focus on improving balance, strength, flexibility, endurance, and overall fitness.

AVGs using various consoles have been demonstrated to successfully promote increased physical activity levels among a variety of age groups [1-6], and to increase physical activity and improve balance and mobility among people with physical disabilities [7-16]. The Wii Fit Balance Board has been used for a variety of outcomes (such as balance training, gait training, physical fitness, and range of motion) and with various populations, such as those with multiple sclerosis, Parkinson disease, incomplete spinal cord injury, and after a stroke [7,8,10-13,17-28]. However, due to accessibility barriers of the Wii Fit Balance Board, none of the studies have included individuals who use a wheelchair as their primary mode of mobility or individuals with complete spinal cord injury. Studies have noted concerns such as lack of safety, intimidation, and worries about falling for individuals with more severe symptoms and less functional ability [19,28].

The limited research has focused on increasing the accessibility of standard video games, with an emphasis on visual disabilities [29-32]. However, little if any research has examined the accessibility of controllers for AVG play or, more specifically, balance board gaming controllers.

### Limitations of the Wii Fit Balance Board

The need to design an adapted version of the Wii Fit Balance Board was motivated by an earlier study conducted by our research team [12] and recommendations from the literature [33]. The previous study involved participants with mobility impairments (N=16) attempting to play 4 AVGs on each of 3 gaming systems. All sessions were video recorded, and study staff noted the barriers faced by the participants in using the various controllers. Based on a review of video recordings and study notes [12], the most limiting features of the off-the-shelf (OTS) Wii Fit Balance Board for successful AVG play by participants with mobility impairments were the following: (1) the Wii Fit Balance Board was completely inaccessible to wheelchair users, (2) it had a small platform area (49.5 cm wide by 30.5 cm deep), (3) it was incompatible with the use of stabilization assistance devices (ie, walker, cane), (4) it required a full range of center-of-balance (COB) motion for responsive game play, and (5) it required the ability to shift the center of gravity in all directions (ie, full range of trunk motion).

**Figure 1:**
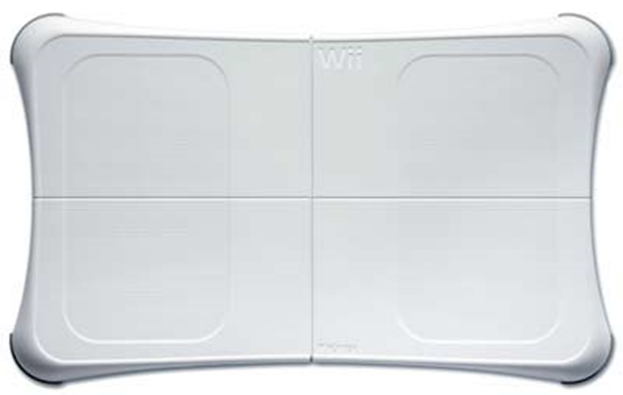
Commercial off-the-shelf Wii Fit Balance Board.

As noted, the biggest limitation of the OTS Wii Fit Balance Board is that it requires participants to be able to stand, thus making the system completely inaccessible to wheelchair users (Figure 1). In addition, given the small size of the platform, it can only accommodate a stance that is no more than shoulder-width apart for most people. Because the board uses load cells to detect the player’s weight and COB shifts, all of the player’s weight must bear on the top of the platform. This limitation makes the board less responsive to those who require the use of stabilization assistance devices (eg, cane, walker) that bear on the floor around the platform. Furthermore, to achieve success, many games require that players have a full range of trunk motion.

### Study Objectives

The aims of this study were to (1) adapt the Wii Fit Balance Board to improve accessibility, and (2) evaluate the usability of the OTS and adapted Wii Fit Balance Board in persons with physical disabilities, specifically mobility impairments.

## Methods

### Adaptation of the Wii Fit Balance Board

#### Adaptations to Improve Accessibility

We presented the above-identified limitations to our engineering design team. We proposed a variety of solutions, and finally decided to make the following mechanical and electrical adaptations to increase the level of accessibility: (1) construct a larger platform area (101.6 cm wide by 96.5 cm deep), (2) build in lateral stabilization supports (ie, handrails), (3) adjust the sensitivity for COB response, and (4) add an accessible wheelchair ramp compliant with the Americans with Disabilities Act (ADA). We selected these adaptations not only to enable wheelchair users to access the Wii Fit Balance Board, but also to make it a universal device with enhanced safety for all users.

#### Product Development

**Figure 2:**
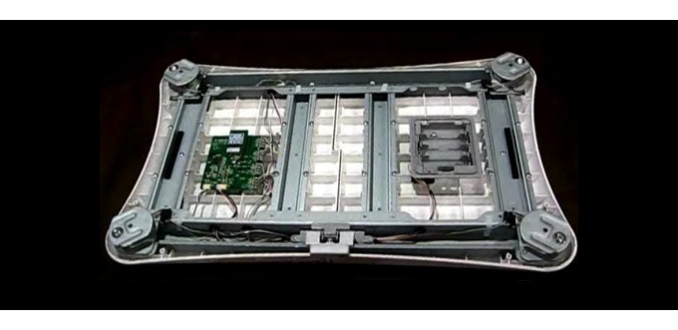
Wii Fit Balance Board electrical components.

We deconstructed the OTS Wii Fit Balance Board so that the electrical components could be reconfigured and integrated into the new form factor and electrical design of the platform. As Figure 2 shows, we removed the bottom cover of the board to access the core electrical components: the electronic control unit (ECU), the battery housing with built-in sync button, the 4 load cells, and the power button. The 4 load cells, wired directly to the ECU, detect the weight and COB of the player. The sync button enclosed in the battery housing enables the user to initiate a Bluetooth link between the board and a Wii system. The power button triggers the ECU to power up and establish a Bluetooth link to the Wii system and to begin transmission of weight and COB data.

**Figure 3:**
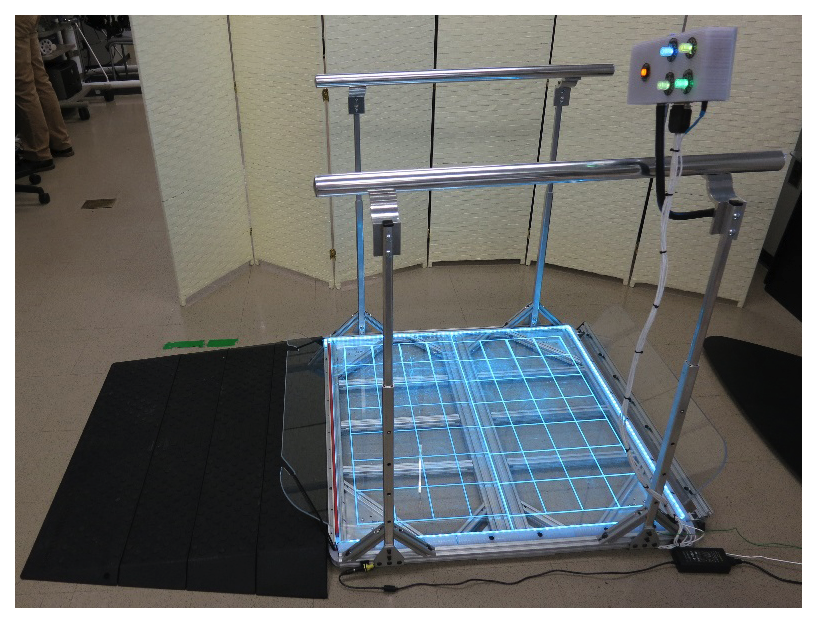
Adapted Wii Fit Balance Board with added ramp, adjustable-height handrails, and control box mounted on the right-side handle.

We focused the mechanical redesign (Figure 3) on providing a larger platform for players to use in both seated and standing positions. To facilitate wheelchair users, we designed the usable space on the platform to be 91.5 cm by 91.5 cm, with the load cells placed at each of the 4 corners. To frame the platform, we used 1-inch t-slot aluminum extrusions, with 2 transparent acrylic sheets (45.7 cm by 91.5 cm by 1.27 cm) for the platform’s surface. To provide the user with an alignment grid, the acrylic sheets were laser etched and edge lit with a variable-color light-emitting diode (LED) strip. This alignment grid provided a guide for proper positioning of a seated player. This alignment is crucial for successful game play, as any major offset to one side of the board causes the player to experience a proportional bias, thereby negatively affecting accurate manipulation of the system.

We purchased an OTS modular ADA-compliant ramp to provide safe access to the top of the platform at a height of 6.35 cm. We designed transparent acrylic roll-off guards to prevent a player using a wheelchair from inadvertently rolling off the front or back of the platform. The front guard had an adjustable hinge that allowed for customized positioning of the guard so as to not interfere with the player’s wheelchair footrests, which can vary widely from one wheelchair configuration to another. The back guard was removable and was dropped into the rear guard slot after the player had positioned himself or herself onto the platform, and was tethered in place with the provided elastic bands.

We integrated ADA-compliant, adjustable-height handrails (Figure 3) into the design to provide lateral stability and additional leverage for players both in seated and in standing positions. The handrails were firmly anchored directly into the platform frame to ensure all of the player’s weight and COB would be accurately captured. The handrails featured 4 telescoping vertical support members that allowed the handrails to be set at different heights (between 62.2 cm and 94 cm), which are within height requirements for ADA-compliant handrails. The rails themselves consisted of 2 ADA-compliant 1.25-inch polished aluminum rails with end caps and ADA-compliant brackets that ensured a continuous grip along the entire length of the rails.

**Figure 4:**
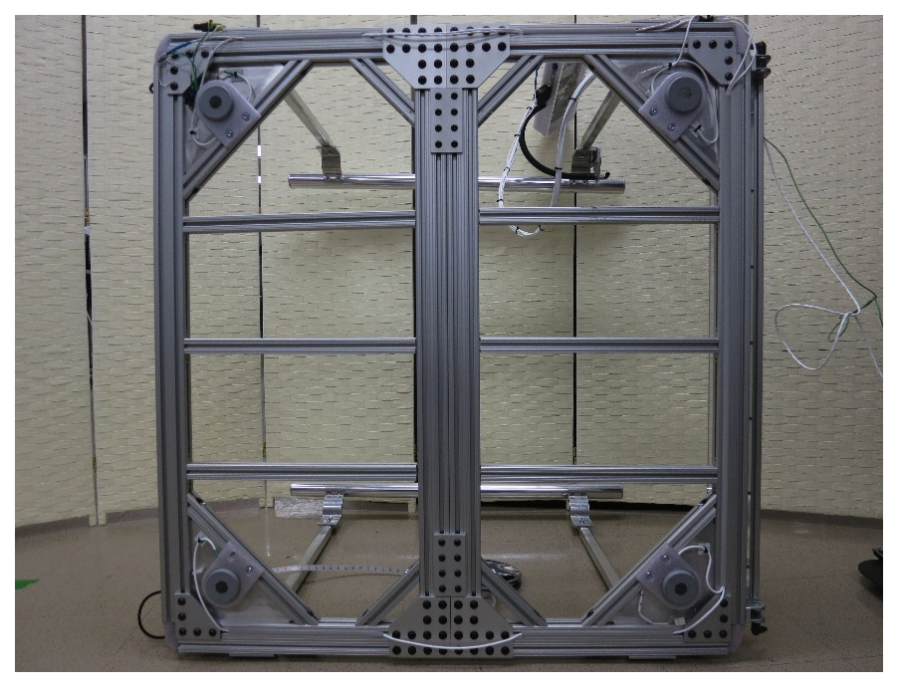
Bottom view of the adapted Wii Fit Balance Board braced for rigidity.

All of the t-slot aluminum extrusions were held together with a combination of t-slot anchors and strategically located bracing plates (Figure 4) to provide the most rigid platform possible. Rigidity is an important feature for use of a low-profile platform that incorporates load cells, because all of the player’s weight must pass through the load cells to the floor for an accurate COB to be calculated, thereby requiring that no part of the frame can deflect under a load such that it contacts the floor. As with the OTS Wii Fit Balance Board, we designed the platform to work best with players who have a gross weight (ie, player plus wheelchair) of less than 150 kg (330 lb).

**Figure 5:**
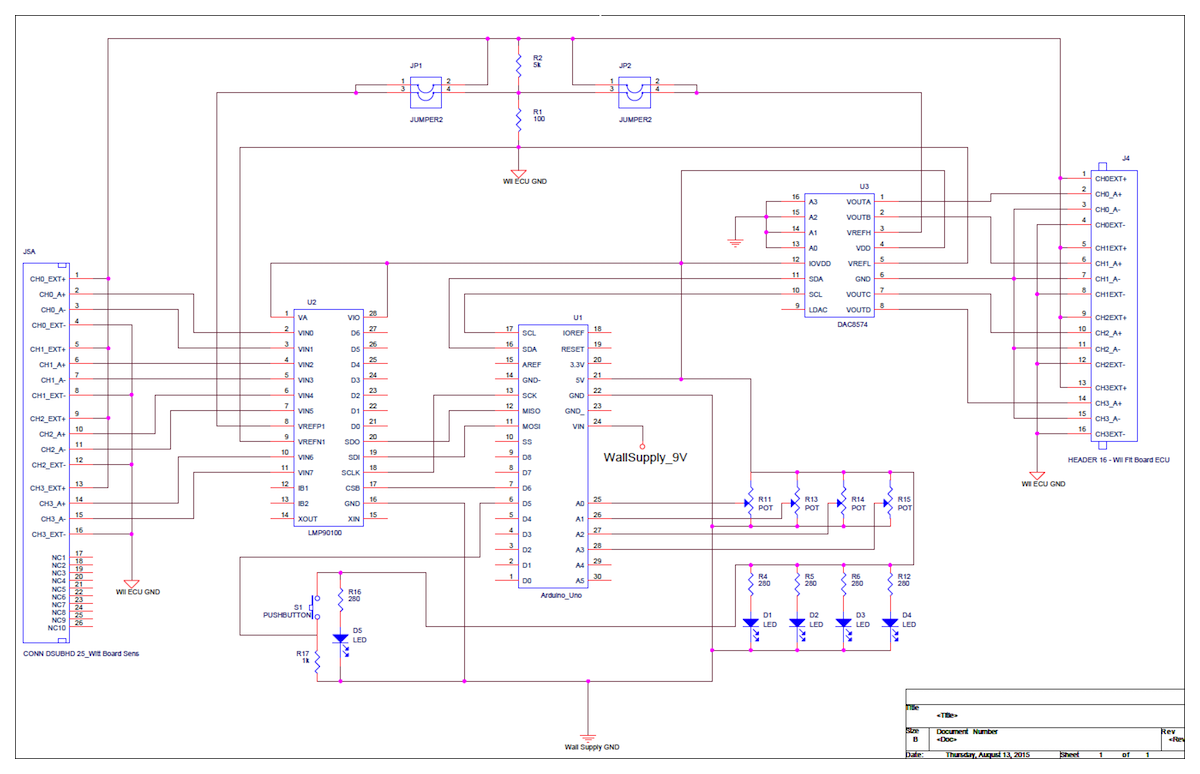
Custom electronics schematic for the adapted Wii Fit Balance Board enabling voltage readings from the load cells to be captured, analyzed, manipulated, and retransmitted to the electronic control unit.

The electrical redesign (Figure 5) required that the voltage readings from the load cells be captured, analyzed, manipulated, and retransmitted to the Wii Fit Balance Board ECU. The redesign provided full control over the data coming from the load cells and consequently allowed for complex functions, such as increased or decreased sensitivity to COB shifts and inclusion of a “jump” button, to be implemented in the firmware algorithm. The electrical components required to accomplish the desired implementation included a 4-channel Texas Instruments LMP09100 analog front-end integrated circuit (Texas Instruments Incorporated, Dallas, TX, USA), an Arduino Uno microcontroller (Arduino, San Jose, CA, USA), and a 4-channel Texas Instruments digital-to-analog converter. The analog front-end integrated circuit provided a 4-channel 24-bit analog-to-digital converter with associated hardware filtering to deliver precision digital measurements of the load cell voltages to the microcontroller over a 4-wire serial peripheral interface bus. The 4-channel 16-bit converter allowed the microcontroller to precisely control (over an interintegrated circuit bus) the manipulated load cell voltages presented to the Wii Fit Balance Board ECU for processing of the COB (and eventual transmission to the Wii system). The microcontroller executed the firmware algorithm that controlled all aspects of the adapted Wii Fit Balance Board, which primarily required the acquisition, manipulation, and simulation of the load cell data at approximately 47 Hz. The Wii Fit Balance Board ECU’s 3.3-V voltage supply was used as the excitation voltage source for the load cells, and a voltage divider was used to scale the 3.3-V full-scale 16-bit digital-to-analog converter voltage down to the 0- to 15-mV range for presentation to the ECU for COB calculation.

We also included in the electrical redesign a control box mounted on a flexible arm that was attached to the right-side handrail (Figure 3). This box housed all of the board’s required electronics and had 4 illuminated dials, 2 illuminated push buttons, and the Bluetooth sync button. A dial labeled “Sensitivity” allowed the user to adjust the balance board sensitivity to shifts in COB. With the dial positioned at 12 o’clock, the sensitivity was at a nominal setting, meaning it was approximately the same as it would be for an OTS Wii Fit Balance Board. With the dial positioned at approximately 7 o’clock, the sensitivity was twice that of the nominal, meaning that any shift in the player’s COB would be greatly exaggerated. With the dial set at approximately 5 o’clock, the sensitivity was zero, meaning that the COB was locked in the neutral or center position. The dial could be set anywhere in between these values in real time during game play. The 2 dials on the lower half of the control box were used to adjust color and brightness on the LED alignment grid strip. The top right unlabeled dial allowed for the player to freeze the output of the platform to allow for more time to position themselves correctly on top of the platform during the in-game calibration sequences. The push button labeled “Jump” allowed the player to perform a jump action in the game without requiring the player to physically jump in place. The blue push button located on the top of the box toggled the Wii Fit Balance Board ECU power and Bluetooth connection.

In accordance with user-centered design principles [34], we focused this project on usability throughout the entire development process. To allow for this continual, systematic evaluation of usability, we developed the adapted controller iteratively and incrementally, sought input from stakeholders at regular intervals, built and refined multiple prototypes incrementally, and involved a multidisciplinary team.

### Usability Testing

#### Design and Setting

All research study procedures were approved by the University of Alabama’s Institutional Review Board. The trial is registered with ClinicalTrials.gov (NCT02994199). Data collection took place at Lakeshore Foundation (Birmingham, Alabama, USA) Exercise and Sport Science Laboratory, which houses a variety of equipment dedicated to comprehensive health promotion and sport science research. For the purposes of this study, participants came to the laboratory for 3 visits. Our protocol article described the study procedures in detail [30].

#### Participants

We included participants (age 10 to 60 years) in the study if they had a confirmed diagnosis of lower-extremity mobility limitation (eg, spina bifida, cerebral palsy, muscular dystrophy, 1 year following spinal cord injury, multiple sclerosis, stroke, or limb loss) with partial or full use of their upper extremities and use of an assistive device (eg, cane, walker, or wheelchair) or problems with gait, balance, or coordination. Participants were excluded if they had an unstable cardiovascular condition, a visual impairment that interferes with playing video games (eg, complete blindness; inability to read game commands on a 52-inch television screen from a distance of 10 feet), or weighed over 150 kg (330 lb) including their assistive device. Following distribution of a flyer at a large health and wellness facility for people with disabilities, the project recruitment coordinator answered calls from or met with interested individuals. At that time, the recruitment coordinator reviewed the inclusion and exclusion criteria with them using a screening form to determine whether they were eligible to participate.

#### Measures

We quantitively evaluated usability of the OTS and adapted versions of the Wii Fit Balance Board using the System Usability Scale (SUS) [35]. The SUS is a simple, 10-item Likert scale, giving a global view of subjective assessments of usability. Various studies have shown that the SUS is a highly robust and versatile tool for usability professionals [36]. The SUS produces a score ranging from 0 to 100, which can be compared with the reported average SUS score of 68, to produce normalized percentile scores [37]. The SUS is generally used after the respondent has had an opportunity to use the system being evaluated, but before any debriefing or discussion takes place [35].

We also recorded each participant’s physical function assessment, health history, video game play history, and assistive device use. For assessment of physical function, each participant performed a series of upper- and lower-body functional movement tasks from the *International Classification of Functioning, Disability and Health* (ICF) [38,39]. Participants completed each task individually and were scored by research staff according to the level of difficulty exhibited in completing the task. As defined in the ICF manual, the scoring was as follows: 0=no difficulty, 1=mild difficulty, 2=moderate difficulty, 3=severe difficulty, and 4=complete difficulty. Two trained research staff independently scored each participant and then reviewed the scores together, if needed, until they reached a consensus for the final score. Video recordings were available for any significant discrepancies. For this project, we added the scores from 10 specific ICF tasks, which assessed lower-extremity function and trunk control, together to create a composite score (ranging from 0 to 40) that we included in the analyses. The individual ICF mobility activities were Squatting, Sitting, Standing, Bending, Shifting the body’s center of gravity, Kicking, Walking short distances, Walking other (marching in place), Running, and Jumping. A low score indicated higher functional ability.

#### Sample Size

The literature suggests that a sample size of 20 is sufficient for detecting usability issues as measured with the SUS [40]. To determine usability of the adapted Wii Fit Balance Board, we asked a random subset of participants (n=25) from our larger study [41] to complete the SUS.

#### Data Analysis

We computed participants’ SUS scores for the OTS and adapted versions of the Wii Fit Balance Board according to the scale’s scoring rubric. Following computation of the scores, we conducted a paired-samples *t* test (IBM SPSS Statistics 22.0, IBM Corporation) to compare the usability scores of the Wii Fit Balance Board with and without adaptation. We also computed the Pearson correlation coefficient between the adapted Wii Fit Balance Board SUS scores and the participant’s lower-extremity function and trunk control score.

## Results

**Figure 6:**
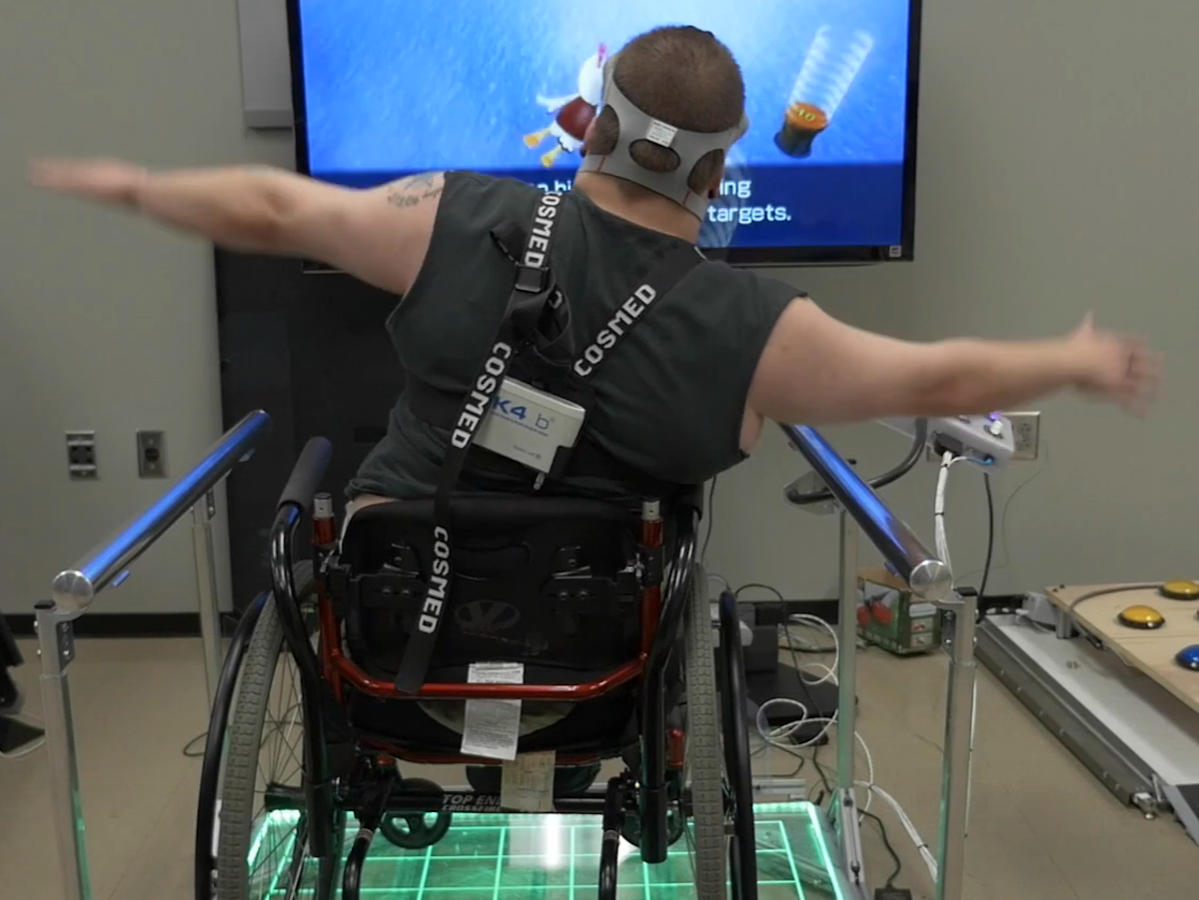
Wheelchair user using adapted Wii Fit Balance Board.

Multimedia Appendix 1 presents the participants’ (n=25) characteristics. The mean age of the participants was 40.16 (SD 14.5) years, with slightly over half of the sample (n=14) being male. They had a variety of conditions: multiple sclerosis (n=6), spinal cord injury (n=5), cerebral palsy (n=4), stroke (n=3), and various other physical impairments (n=7). The primary mode of mobility was manual wheelchair (n=9), no assistive aid (n=11), walker (n=2), and cane, leg brace, and prosthetic leg each used by 1 participant. A total of 15 participants had prior AVG experience, with only 2 having had prior experience with the Wii Fit Balance Board. Figure 6 shows a participant on the modified Wii Fit Balance Board and Multimedia Appendix 2 presents a video of participants using the Wii Fit Balance Board.

Among the sample, half of the participants (n=13), all assistive aid users, could not use the OTS version of the Wii Fit Balance Board. The mean SUS score for the OTS Wii Fit Balance Board among the participants who could use it (n=12) was 66.88 (SD 20.14). All participants (n=25) were able to use the adapted version of the Wii Fit Balance Board, resulting in a mean SUS score of 71.7 (SD 18.03). The mean SUS score for the adapted Wii Fit Balance Board among the participants who could use the OTS version (n=12) was 67.08 (SD 21.76), whereas the mean was 75.96 (SD 13.24) among the participants (n=13) who could not use the OTS version. Table 1 shows participants’ individual SUS scores for the OTS and adapted versions of the Wii Fit Balance Board.

We found a significant Pearson correlation (*r*=.692, *P*<.001) between the difference in SUS scores (OTS, adapted) and the physical function scores.

**Table 1:** System Usability Scale (SUS) scores for each participant.

Participant ID	OTS^a^ Wii Fit Balance Board SUS score	Adapted Wii Fit Balance Board SUS score
1	75	75
2	57.5	62.5
3	0	77.5
4	50	37.5
5	100	37.5
6	100	100
7	60	82.5
8	42.5	70
9	50	55
10	57.5	100
11	0	62.5
12	0	97.5
13	0	75
14	0	100
15	0	70
16	0	65
17	0	70
18	92.5	45
19	0	55
20	55	57.5
21	0	70
22	62.5	82.5
23	0	90
24	0	75
25	0	80

^a^OTS: off-the-shelf.

## Discussion

### Principal Findings

A first step in determining a system’s success or failure is to evaluate its usability [33]. SUS computed scores range from 0 to 100, but do not equate to percentages. As a way to interpret SUS scores, Bangor et al established an adjective rating scale to correspond to the computed scores [36]. Based on the adjective scale, the adapted Wii Fit Balance Board’s score of 71.7 among all participants translates to “good” and belongs to the acceptable range of SUS scores. Additionally, among participants who could not use the OTS Wii Fit Balance Board, the adapted Wii Fit Balance Board had a mean SUS score of 75.96, which translates to “excellent”. For those who could use it, the SUS score for the OTS Wii Fit Balance Board was 66.88 and translates to “good”.

The results of this study demonstrate that employing user-centered design principles can increase accessibility and safety of AVG controllers, resulting in increased usability, especially for individuals who require an assistive aid for mobility. The increased usability of the adapted Wii Fit Balance Board is the result of a user-driven design, where users provided initial feedback to help in the design of the first prototype. Subsequent user feedback at multiple levels throughout the design process resulted in a highly usable product. The most significant benefit offered by the adapted Wii Fit Balance Board is that it doubled the number of the users able to engage in AVG play on the Wii Fit Balance Board, and adaptation of the Wii Fit Balance Board was rated as excellent. Furthermore, the strong correlation between the difference in usability scores and physical function score suggests that the Wii Fit Balance Board adaptations were increasingly useful (highly usable) for participants with a stronger need (ie, increased lower-extremity function and trunk control limitations).

We observed a few exceptions to the generally positive rating of the adapted Wii Fit Balance Board. For instance, 1 participant (ID #18), who did not use an assistive aid, demonstrated high physical function and had prior experience with the Wii Fit Balance Board, rated usability of the OTS version higher than that of the adapted board. Similarly, another participant (ID #5) scored usability of the OTS Wii Fit Balance Board much higher than the adapted version. This large difference in perception of usability is perhaps explained by the fact that the participant had previously played AVGs using the Wii Fit Balance Board and perceived her game play performance using the OTS controller as proficient given her high level of lower-extremity function with use of a prosthetic leg. This participant likely perceived the adapted board as having little value and perhaps viewed it as an unnecessary adaptation. Had the participant conducted the trials without her prosthetic leg, however, we would expect that she would have rated the usability of the adapted board more highly. As noted in one study, individuals with unilateral transtibial amputation who used a custom-fit prosthetic leg were able to perform similarly to individuals without amputation on a variety of physical performance measures [42].

This study aimed to evaluate the usability of the adapted Wii Fit Balance Board; however, generalizability of the results is limited by the fact that a large variety of users, with different types of impairments and assistive aids, were included in the sample. A further stratified study, evaluating the usability of the device qualitatively and quantitatively within selected population groups, such as those with specific degrees of mobility impairment or using an assistive aid, would help identify a much more precise level of usability for this adapted gaming controller. Furthermore, the prototype evaluated in this study was based on the commercial Wii Fit Balance Board and thus was limited to a maximum weight of user and wheelchair (if needed) to 150 kg (330 lb).

One addition to the adapted Wii Fit Balance Board that we did not evaluate in the context of this study was a button to adjust the sensitivity of the controller. This addition was based on the range of movements required for various games, from extreme whole-body movements to very limited and controlled small-segment (eg, hand) movements. The sensitivity control was introduced to counter these requirements and enable participants with limited functional movement to successfully engage in AVG play. Further investigation should include evaluation of this feature and its effect on usability of the system.

Individuals with disabilities experience high rates of secondary conditions, with lack of physical activity being an important contributing factor [43,44]. Participation in AVGs has been shown to increase physical activity in people with disabilities [1-5,17,18]. Our study successfully designed, engineered, and evaluated the removal of the access barrier to a widely used gaming controller (Wii Fit Balance Board), thereby enabling users with a range of physical function to play a variety of games that can be controlled through the Wii Fit Balance Board.

To date, several studies have examined usability of AVG controllers with a focus on older adult populations most often playing researcher-developed games [45,46]. Our study was unique, in that it evaluated the usability of a mainstream AVG controller (Wii Fit Balance Board), redesigned with major hardware adaptations to enhance the accessibility, safety, and user experience for individuals with impaired mobility during play of commercially available games. The results demonstrated that application of user-centered design principles to the adaptation of gaming controllers can result in acceptable and usable AVG controllers for a wide range of individuals with varying physical disabilities. Future efforts should be directed toward user-centered design and adaptation of other OTS AVG controllers with subsequent evaluation of usability and acceptability. It is important that all AVG controllers be designed universally, or be adapted, to meet the needs of people with various physical disabilities and provide them with equal opportunities to engage in AVG play. Although the results of this study demonstrated the usability of the adapted Wii Fit Balance Board, its effect on the level of physical activity and other important fitness and health outcomes is an important next step. We are engaged in a preliminary study to evaluate energy expenditure during AVG play on the adapted Wii Fit Balance Board in persons with physical disabilities, specifically those with mobility impairments.

### Conclusions

This study demonstrated a successful adaptation of the Wii Fit Balance Board, thereby enabling AVG play as an option for individuals with mobility impairments. Evaluation of the usability of the OTS and adapted versions of the Wii Fit Balance Board showed that users with various impairments, including those who use a wheelchair for mobility, were able to engage in AVG play using the Wii Fit Balance Board as a result of the adaptation. Additionally, results suggest that, the less lower-extremity function and trunk control participants have, the more highly they rate the usability of the adapted Wii Fit Balance Board. Future studies should consider adaptation of other available AVG controllers following a similar user-centered design approach.

## Appendix

Multimedia Appendix 1 Participant characteristics (Table).

Multimedia Appendix 2 Participants using the adapted Wii Fit Balance Board (Video).

## Abbreviations

ADA: Americans with Disabilities Act
AVG: active video game
COB: center of balance
ECU: electronic control unit
ICF: International Classification of Functioning, Disability and Health
LED: light-emitting diode
OTS: off-the-shelf
SUS: System Usability Scale
