# Supplementary material for: Adapting the Wii Fit Balance Board to Enable Active Video Game Play by Wheelchair Users: User-Centered Design and Usability Evaluation
Source: JMIR Rehabil Assist Technol. 2018 Mar 6;5(1):e2. doi: 10.2196/rehab.8003 (PMC5861301; doi:10.2196/rehab.8003)
Supplement: Multimedia Appendix 1 [file rehab_v5i1e2_app1.pdf]

Participant Characteristics

ID	Age	Sex	Condition	Assistive Device	AVG Experience	Wii Fit Experience	Total Lower Body Function Score*
1	34	M	Cerebral Palsy	None	No	No	4
2	57	F	Multiple Sclerosis	None	Yes	No	0
3	40	F	Multiple Sclerosis	Walker	No	No	0
4	29	M	Traumatic brain injury	None	Yes	No	6
5	51	F	Amputee – single below knee	Prosthetic leg	Yes	Yes	0
6	31	M	Traumatic brain injury	None	Yes	No	0
7	60	M	Stroke	None	No	No	0
8	40	F	Multiple Sclerosis	None	Yes	No	5
9	58	M	Stroke	None	No	No	13
10	59	F	Stroke	None	Yes	No	1
11	38	M	Spina Bifida	Manual Wheelchair	Yes	No	30
12	42	M	Spinal Cord Injury	Manual Wheelchair	Yes	No	32
13	33	M	Spinal Cord Injury	Manual Wheelchair	Yes	No	37
14	27	F	Spinal Cord Injury	Manual Wheelchair	Yes	No	33
15	46	M	Mobility Impairment	Walker	Yes	No	25
16	58	F	Spinal Cord Injury	Cane	No	No	16
17	46	M	Spinal Cord Injury	Manual Wheelchair	No	No	33
18	39	F	Multiple Sclerosis	None	Yes	Yes	0

19	18	M	Cerebral Palsy	Manual Wheelchair	No	No	31
20	58	F	Multiple Sclerosis	None	No	No	0
21	14	M	Ataxia	Manual Wheelchair	Yes	No	34
22	47	F	Multiple Sclerosis	None	No	No	0
23	47	F	Morbid Obesity	Manual Wheelchair	Yes	No	24
24	19	M	Cerebral Palsy	Manual Wheelchair	No	No	32
25	13	M	Cerebral Palsy	Leg Brace	Yes	No	1

*Details regarding this assessment can be found in: Malone LA, Padalabalanarayanan S, McCroskey J, Thirumalai M. Protocol for assessment of active video gaming using adapted controllers by individuals with physical disabilities. JMIR Res Protoc 2017 Jun 16;6(6):e116. PMID: 28623186
